# Prevalence of obesity and body size perceptions in urban and rural Senegal: new insight on the epidemiological transition in West Africa

**DOI:** 10.5830/CVJA-2017-034

**Published:** 2017

**Authors:** Enguerran Macia, Emmanuel Cohen, Gilles Boetsch, Lamine Boetsch, Emmanuel Cohen, Priscilla Duboz

**Affiliations:** Faculty of Medicine, Pharmacology and Odontology, University of Cheikh Anta Diop, Dakar, Senegal; National Centre for Scientific Research, University of Bamako, Mali; and National Centre for Scientific and Technological Research, Burkina Faso; Faculty of Medicine, Pharmacology and Odontology, University of Cheikh Anta Diop, Dakar, Senegal; National Centre for Scientific Research, University of Bamako, Mali; and National Centre for Scientific and Technological Research, Burkina Faso; Faculty of Medicine, Pharmacology and Odontology, University of Cheikh Anta Diop, Dakar, Senegal; National Centre for Scientific Research, University of Bamako, Mali; and National Centre for Scientific and Technological Research, Burkina Faso; Faculty of Medicine, Pharmacology and Odontology, University of Cheikh Anta Diop, Dakar, Senegal; National Centre for Scientific Research, University of Bamako, Mali; and National Centre for Scientific and Technological Research, Burkina Faso; Department of Eco-Anthropology and Ethnobiology, National Museum of Natural Science, University of Paris, France; Department of Anthropology, Ethics and Health, Santé, Aix-Marseille University, Marseille, France

**Keywords:** Africa, biological anthropology, epidemiological transition, nutrition transition, overweight

## Abstract

**Background:**

The objectives of this study were to assess the prevalence of obesity in Dakar and in Tessekere, a rural municipality in northern Senegal, and to compare ideal body size between these populations.

**Methods:**

A cross-sectional survey was carried out in 2015 on a representative sample of 1 000 adults, aged 20 years and older in Dakar, and 500 adults of the same age in Tessekere.

**Results:**

The prevalence of obesity and overweight was higher in Dakar than in Tessekere. However, overweight and obesity rates of young women living in this rural area were close to those of young women in Dakar. At a body mass index of 27.5 kg/m², less than 40% of the men in Dakar and Tessekere found themselves too fat, compared to 50% of urban women and 30% of rural women.

**Conclusion:**

This study explains how and why obesity is becoming a rural health problem in Senegal.

## Introduction

Overweight and obesity are important risk factors for cardiovascular disease.1,2 The increasing prevalence of obesity during the last few decades in a number of countries^3^ has been reported as a global pandemic and a major public health issue worldwide.^4^-^07^ Sub-Saharan Africa (SSA) is not immune to this epidemic.^8^,^9^ In urban West Africa, the prevalence of obesity more than doubled from 7.0% in 1990–94 to 15.0% in 2000–04.10 However, over this 15-year period, Abubakari and colleagues noted that obesity rates seemed to remain unchanged in rural West Africa, possibly due to the small number of studies retrieved from these populations.^10^

Despite the threat posed by obesity in West Africa, there are very few studies addressing this issue in Senegal and none in the rural areas. To our knowledge, few studies have evaluated the prevalence of obesity among both men and women in Dakar,^11^ the political and economic capital of the country. In terms of body mass index (BMI), the prevalence of overweight and general obesity in 2009 was 22.3 and 8.3%, respectively, in Dakar, whereas using waist circumference (WC), the prevalence of central obesity was 21.2%.^11^ Only by monitoring prevalence over time can the evolution of the obesity epidemic in the Senegalese capital be understood.^1^

Various factors contribute to the high prevalence of obesity in SSA.^8^,^9^ More precisely, numerous macrosocial (e.g. urbanisation,12 globalisation^9^), genetic,^13^ behavioural (mainly diet and physical activity^14^), sociodemographic,15 and culturally underlying16 factors have been reported as determinants of obesity in West Africa. In the context of this comparative urban–rural study in Senegal, a focus on urbanisation, sociodemographics and perception of body size is fundamental.

It is now well established that urbanisation is a major driving force in obesity, by reducing physical activity and increasing consumption of energy-dense diets.^17^ In West Africa, urban residents have three times the odds of being obese than rural residents.^10^ Among sociodemographic factors, age and gender have regularly been shown to be associated with obesity in SSA8 and West Africa.^10^

Beyond these recurrent and robust predictors, the role of socio-economic status (SES) seems more complex in SSA. Indeed, while studies regularly report that obesity is significantly more likely to occur in the highest SES group,^8^ Ziraba and colleagues observed that the increase in obesity was higher among the poorest than among the richest African urban dwellers during the period 1995 to 2005.^18^ In line with the epidemiological transition occurring in SSA,^19^,^20^ the relationship between obesity and SES is likely to change in the coming years and gradually affect the lowest SES groups more than the highest.

In SSA, positive traditional representations of stoutness – the social validation of the big belly for men and large hips for women^21^ – may also contribute to the gradual development of obesity. Obesity is a concept that is viewed differently across cultures.^22^ In SSA, where HIV and other diseases associated with wasting away are prevalent, overweight and obesity have been associated with health.^16^,^23^ Moreover, once married, extra weight is seen as an indicator that the spouse is well cared for.^24^ In Pikine, a suburb of Dakar, these positive perceptions of stoutness have been observed among women.^25^ However, no study has been conducted from this perspective among urban men, or among the rural population.

Therefore, the objectives of this study were (1) to assess and compare the prevalence of obesity, general and central, in Dakar and in Tessekere, a rural municipality in northern Senegal, and to analyse trends in obesity in Dakar; (2) to determine sociodemographic risk factors for obesity in both environments; and (3) to compare ideal body size between urban and rural areas.

## Methods

The study was approved by the National Ethics Committee for Health Research of Senegal (protocol SEN13/67, no 0272). The research was conducted in accordance with the Declaration of Helsinki, and written informed consent was obtained from participants.

This study was conducted from February to August 2015 on a sample of 1 000 individuals, aged 20 years and older in Dakar, and on a sample of 500 adults of the same age bracket in the Tessekere municipality. The samples were constructed using the combined quota method (cross-section by age, gender and town of residence in Dakar; only by age and gender in Tessekere municipality) in order to strive for representativeness of the population aged 20 years and older living in the department of Dakar and in Tessekere municipality. Data from the Agence Nationale de la Statistique et de la Démographie dating from the last census (2013) were used.

The quota variables used were gender (male/female), age (20–29, 30–39, 40–49, 50–59, and 60 years and over, with an upper age limit of 100 years) and, for Dakar, town of residence. In Dakar, the towns were grouped by the four arrondissements making up the department: Plateau-Gorée (five towns), Grand Dakar (six towns), Parcelles Assainies (four towns) and Almadies (four towns). In practical terms, this method requires constructing a sample that reflects the proportions observed in each target population. For example, according to the last census, men aged 20–29 years living in the town of Medina (arrondissement of Plateau-Gorée) represented 1.9% of the population aged 20 years and over living in the department of Dakar. The sample was constructed to reflect this proportion and it included 19 men aged 20–29 living in this town.

Inclusion criteria were individuals 20 years old or older, living in the department of Dakar. Pregnant women were excluded from the study.

Eight trained investigators (PhD students in Medicine, Pharmacy and Sociology) started out each day from different points in each town (Dakar) or camp (Tessekere) to interview individuals in Wolof, Haalpulaar or French in every third home. Investigators had a certain number of individuals to interview to meet the quotas. Only one person was selected as a respondent in each home. Investigators went to the house, inquired aboutthe inhabitants and then chose the first person they saw who met the characteristics needed for the quotas. In-person interviews were conducted. They ranged from 45 minutes to more than one hour and 30 minutes, depending on respondent availability and desire to talk.

Weight was measured using a digital scale (measurement accuracy of 100 g), with subjects dressed in minimal clothing and barefoot. To measure height, the subject was to stand ‘at attention’, arms at the sides, heels together, without shoes. Following World Health Organisation (WHO) recommendations, BMI was calculated by dividing the weight (kg) by the square of the height (m^2^). Underweight was defined as BMI < 18.5 kg/m^2^; overweight was defined as 25 ≤ BMI < 30 kg/m^2^; and obesity corresponded to a BMI of ≥ 30 kg/m^2^.^26^

Waist circumference (WC) was measured at the narrowest point of the abdomen at the end of a normal expiration. WC was measured using a measuring tape with 1-mm accuracy. WC of ≥ 102 cm in men and ≥ 88 cm in women was considered central obesity.^27^ Waist-to-hip ratio (WHR) was also used as a criterion of central obesity: a WHR of ≥ 0.9 in men and ≥ 0.8 in women was considered central obesity.^28^

Among the sociodemographic data collected during the interviews, three variables were taken into account for this study: age, gender and educational level. Four age groups were defined: 20–29, 30–39, 40–49 and 50 years and over. Gender was coded as follows: 1 for women, 0 for men. In Dakar, five levels of education were defined based on the Senegalese school system: none, primary (one to five years of schooling), intermediate (six to eight years), secondary (nine to 12 years), and university (13 years and over). In the Tessekere municipality, given the large proportion of persons who have never attended school (76%), the educational level was dichotomised: no schooling/one or more years of schooling.

Satisfaction with body weight was assessed in one question, with five possible responses: ‘Do you think you are: too thin, a little too thin, average, a little too fat, too fat?’ To determine ideal body size, we took the BMI at which the same percentage of individuals believed they were too heavy as those who felt they were too thin.^29^

We also used the body size scale (BSS), developed and validated by Cohen et al. in Senegal,30 to assess ideal body size (IBS) of women and men, to obtain a complementary representation of body image assessed from the questionnaire. This tool has two advantages: (1) it consists of a gender-specific scale of nine models; and (2) it represents real black models with their anthropometric characteristics to assess specific body weight perceptions in African populations. One model represents the underweight category, three models the normal-weight category, two models the overweight category, and one model each class of obesity level as defined by the WHO (30.0 < BMI ≤ 34.9 kg/m², 35.0 < BMI ≤ 39.9 kg/m², and ≥ 40 kg/m²). BSS was considered a numerical variable, as each human picture ranged from 1 to 9 according to increasing BMI categories to measure ideal body size.

## Statistical analysis

To answer our research questions, we used the Student’s t-test, ANOVA, chi-squared test and logistic regressions. Results are expressed as mean ± standard deviation for continuous variables or as percentages for categorical variables. Bivariate comparisons were performed using the Student’s t-test, ANOVA for continuous variables, and chi-squared tests for categorical variables. Multivariate analyses were performed using binary logistic regression and results are expressed as odds ratios with 95% confidence intervals (CIs). The software used for the statistical analysis was SPSS Statistics 22 for Windows.

## 

Among the 1 000 individuals included in the Dakar sample, 16 women were excluded because they reported pregnancy. Similarly, four women of the Tessekere sample were also excluded for pregnancy. Analyses were finally performed on a sample of 984 Dakarites and 496 Tessekere dwellers. The distributions of height, weight, BMI, WC, WHR, general and central obesity, sociodemographic variables, and comparisons between males and females in both environments are summarised in Table 1. The results show that men and women differed for all the factors studied except for age in both environments, and for WHR in Dakar.

**Table 1:** Demographic and anthropomatic characteristics of the sample

**	*Dakar*				*Tessekere*			
*Characteristics*	*Total (n = 984)*	*Male (n = 494)*	*Female (n = 490)*	*p-value*	*Total (n = 496)*	*Male (n = 241)*	*Female (n = 255)*	*p-value*
Age (years)	35.70 ± 13.16	35.89 ± 13.27	35.51 ± 13.07	0.652	37.33 ± 15.25	37.26 ± 15.45	37.40 ± 15.08	0.917
Height (cm)	172.56 ± 9.87	178.96 ± 8.07	166.11 ± 6.88	< 0.001	169.63 ± 10.38	175.85 ± 8.09	163.75 ± 8.77	< 0.001
Weight (kg)	69.28 ± 14.44	70.21 ± 16.67	68.34 ± 16.00	0.043	60.25 ± 12.32	62.38 ± 11.26	58.23 ± 12.96	< 0.001
BMI (kg/m^2^)	23.33 ± 4.89	21.91 ± 3.54	24.76 ± 5.59	< 0.001	20.97 ± 4.07	20.15 ± 3.24	21.74 ± 4.60	< 0.001
General obesity, n (%)	95 (9.7)	14 (2.8)	81 (16.5)	< 0.001	14 (2.8)	2 (0.8)	12 (4.7)	0.009
WC (cm)	84.31 ± 13.02	81.51 ± 10.65	87.14 ± 14.51	< 0.001	77.25 ± 10.59	76.13 ± 9.31	78.32 ± 11.59	0.021
Central obesity by WC, n (%)	256 (26)	21 (4.3)	235 (48)	< 0.001	59 (11.9)	3 (1.2)	56 (22.?)	< 0.001
WHR	0.836 ± 0.081	0.837 ± 0.069	0.834 ± 0.092	0.579	0.839 ± 0.079	0.847 ± 0.075	0.831 ± 0.082	0.019
Central obesity by WHR, n (%)	393 (39.9)	83 (16.8)	310 (63.3)	< 0.001	117 (23.6)	17 (7.1)	100 (39.2)	< 0.001
Educational level (Dakar/Tessekere), n (%)								0.006
None/none	208 (21.1)	84 (27)	124 (25.3)		373 (75.2)	168 (69.7)	205 (80.4)	
Primary/1 year or +	348 (35.5)	163 (33)	185 (37.8)		123 (24.8)	73 (30.3)	50 (19.6)	
Intermediate	197 (20)	109 (22.1)	88 (18)						
Secondary	91 (9.2)	51 (10.3)	40 (8.2)					
University	140 (14.2)	87 (17.6)	53 (10.8)					

BMI: body mass index, WC: waist circumference, WHR: waist–hip ratio.

In Dakar, the prevalence of underweight, overweight and general obesity in terms of BMI was 12.6% (95% CI: 10.5–14.7), 19.2% (95% CI: 16.7–21.7) and 9.7% (95% CI: 7.9–11.5), respectively. The prevalence of central obesity was 26.0% (95% CI: 23.3–28.7) using WC, and 39.9% (95% CI: 36.8–43.0) using WHR (Table 2).

**Table 2:** Prevalence (%) of underweight, overweight, general obesity and central obesity by place of residence

*Criterion*	*Category*	*Dakar*	*Tessekere*
BMI	Underweight	12.6 (10.5–14.7)	29.6 (25.6–33.6)
	Overweight	19.2 (16.7–21.7)	13.3 (10.3–16.3)
	General obesity	9.7 (7.9–11.5)	2.8 (1.3–4.3)
WC	Central obesity	26.0 (23.3–28.7)	11.9 (9.1–14.7)
WHR	Central obesity	39.9 (36.8–43.0)	23.6 (19.9–27.3)

BMI: body mass index, WC: waist circumference, WHR: waist-hip ratio. In brackets: 95% confidence limits.

In Tessekere, the prevalence of underweight, overweight and general obesity in terms of BMI was 29.6% (95% CI: 25.6– 33.6), 13.3% (95% CI: 10.3–16.3) and 2.8% (95% CI: 1.3–4.3), respectively. The prevalence of central obesity was 11.9% (95% CI: 9.1–14.7) using WC, and 23.6% (95% CI: 19.9–27.3) using WHR (Table 2).

Dakar residents were more often overweight and obese and less often thin than the Tessekere inhabitants [χ² (3 df) = 80.9; p < 0.001]. Likewise, they showed higher central obesity rates than the Tessekere inhabitants [WC: χ² (1 df) = 39.3, p < 0.001; WHR: χ² (1 df) = 39, p < 0.001].

In Dakar as in Tessekere, bivariate analyses showed that all the sociodemographic factors studied were associated with general and central obesity (Table 3). The prevalence of general and central obesity rose gradually with age in both environments, except for obesity based on WC in Tessekere, which reached its highest rate among people between the ages of 40 and 49 years. In the urban and rural areas studied, general obesity affected women six times more often than men, and their WC exceeded the threshold of obesity 11 times and 18 more often than men in Dakar and Tessekere, respectively.

**Table 3:** Prevalence (%) of underweight, overweight, obesity and central obesity by age, gender and educational level in Dakar and Tessekere

**	*Obesity based on BMI*				*Obesity based on WHR*				*Obesity based on WC*			
*Variable*	*n*	*Underweight*	*Overweight*	*Obese*	*p-value*	*Obese*	*p-value*	*Obese*	*p-value*
Dakar									
Age (years)									
20–29	413	18.6	12.8	3.6	< 0.001	26.2	< 0.001	12.6	< 0.001
30–39	266	11.3	22.6	9.8		37.6		25.9	
40–49	156	5.1	20.5	16.7		54.5		37.2	
≥ 50	149	6	29.5	18.8		67.1		51.7	
Gender									
Male	494	15.4>	14	2.8	< 0.001	16.8	< 0.001	4.3	< 0.001
Female	490	9.8	24.5	16.5		63.3		48	
Educational level									
Illiterate	208	8.7	21.6	12	< 0.001	51.9	< 0.001	32.2	< 0.001
Primary	348	10.9	21.6	10.3		40.8		29.9	
Intermediate	197	13.2	20.8	10.2		35		25.4	
Secondary	91	19.8	15.4	9.9		41.8		25.3	
University	140	17.1	10	3.6		25.7		8.6	
Tessekere									
Age (years)									
20–29	200	33	9.5	0	< 0.001	14	< 0.001	4.5	< 0.001
30–39	115	30.4	17.4	1.7		20		9.6	
40–49	77	27.3	11.7	6.5		28.6		23.4	
≥ 50	104	24	17.3	6.7		42.3		20.2	
Gender									
Male	241	34.4	8.7	0.8	< 0.001	7.1	< 0.001	1.2	< 0.001
Female	255	25.1	17.6	4.7		39.2		22	
Educational level									
None	373	30.6	12.9	2.9	NS	26.5	< 0.001	13.1	NS
1 year and +	123	26.8	14.6	2.4		14.6		8.1	

BMI: body mass index, WC: waist circumference, WHR: waist-hip ratio.

**Fig. 1.**
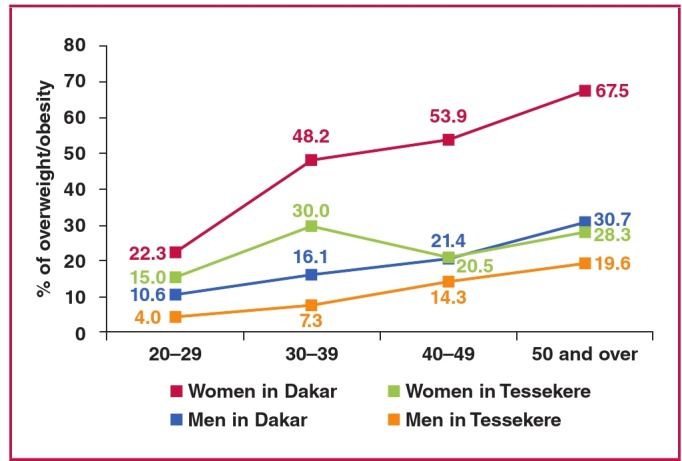
Age- and gender-specific prevalence (%) of overweight/obesity in Dakar and Tessekere.

As shown in Fig. 1, the prevalence of overweight/obesity (using BMI) rose with age among men and women in Dakar. The same pattern was observed among men in Tessekere. However, among rural women, the prevalence of overweight/obesity reached its highest rate between the ages of 30 and 39 years.

Multivariate analyses showed that age and gender were the primary risk factors for overweight/obesity in Dakar and Tessekere (Table 4). Educational level also showed significant associations with BMI ≥ 25 kg/m², but only in the urban area, where people with between one and eight years of schooling had greater chances of being overweight or obese than people who attended university. Gender was the primary risk factor for central obesity (WC and WHR) in both environments (Table 4).

**Table 4:** Adjusted odds ratio (OR) for overweight/obesity and central obesity in Dakar (n = 984) and Tessekere (n = 496)

**	*Overweight/obesity*		*Obesity based on WHR*		*Obesity based on WC*	
*Variables*	*OR*	*95 % CI*	*OR*	*95 % CI*	*OR*	*95 % CI*
Dakar						
Age (20–29)						
30–39	2.39***	1.62–3.52	1.96**	1.32–2.92	2.89***	1.82–4.60
40–49	3.17***	2.03–4.95	5.34***	3.29–8.66	7.47***	4.24–13.18
≥ 50	5.38***	3.42–8.45	12.40***	7.35–20.93	29.51***	14.79–58.90
Gender (men)						
Women	3.85***	2.81–5.29	13.24***	9.21–19.05	49.33***	26.74–91.01
Educational level (university)						
None	1.47	0.80–2.72	1.23	0.70–2.18	1.43	0.65–3.16
Primary	1.85*	1.05–3.26	1.1	10.65–1.85	2.58*	1.24–5.40
Intermediate	1.96*	1.07–3.58	0.94	0.53–1.66	2.58*	1.17–5.68
Secondary	1.59	0.77–3.25	1.59	0.81–3.12	2.91*	1.17–7.21
Tessekere						
Age (20–29)						
30–39	2.35*	1.19–4.65	1.55	0.81–2.96	2.29	0.89–5.89
40–49	2.49*	1.13–5.46	2.53**	1.25–5.13	8.74***	3.34–22.83
≥ 50	3.89***	1.93–7.86	6.03***	3.13–11.60	7.67***	3.02–19.45
Gender (men)						
Women	2.93***	1.71–5.02	10.08***	5.59–18.18	27.16***	8.15–90.55
Educational level (1 year or +)						
None	0.57	0.31–1.05	1.07	0.57–2.00	0.56	0.23–1.34

*p < 0.05; **p < 0.01; ***p < 0.001. BMI: body mass index, WC: waist circumference, WHR: waist-hip ratio.

In Dakar, 50% of the study participants were satisfied with their weight, 27% thought they were too thin and 23% too fat. Men were more often satisfied with their weight than women (57 vs 43%), who in turn more often thought themselves too heavy (33 vs 13%; p < 0.001). In Tessekere, the majority found themselves too thin (53%), 8% believed they were too fat, and 39% were satisfied with their weight. Men were more often satisfied with their weight than women (45 vs 34%; p < 0.01).

**Fig. 2.**
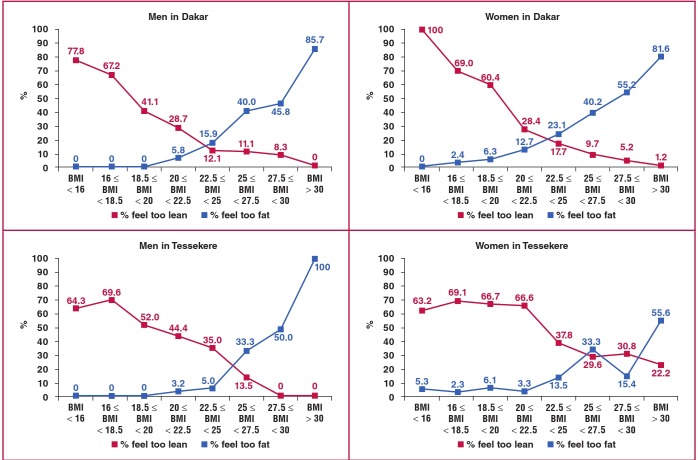
Satisfaction with weight by BMI among men and women in Dakar and Tessekere.

Fig. 2 shows that ideal BMI for men and women in Dakar was found to be 23.5 kg/m². In Tessekere, ideal BMI for men was 25.5 kg/m². For women in this rural area, the tendency was not as clear, but the ideal BMI for rural women could nevertheless be situated in the overweight category. We should note that at a BMI of 27.5 kg/m², only 42% of the men in Dakar felt too fat, as opposed to 49% of the women. In Tessekere, for the same BMI, 41% of the men felt too heavy as opposed to only 30% of the women.

**Fig. 3.**
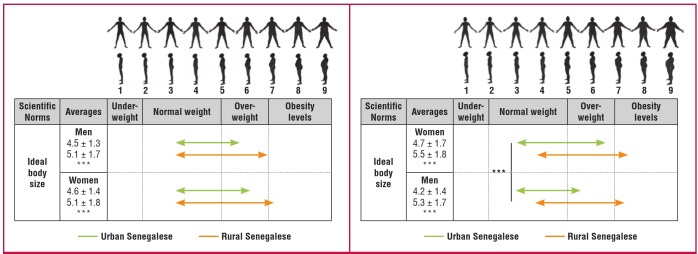
Perceptions of ideal body size on masculine and feminine body size scale.

In Tessekere, 10 people were unable to judge ideal body size by the BSS. Analyses concerning this scale were therefore done on 486 participants in the rural area and 984 in the urban area (Fig. 3). First, we observed that for both male and female scales, averages of IBS for oneself and the opposite sex were lower in urban Senegalese than in rural Senegalese. The ideal male and female bodies fell within the normal range in Dakar, and in the overweight category in Tessekere. Second, there were no significant differences between men and women from each environment on each scale, except for the female scale in Dakar; urban women perceived the ideal female body size as heavier than their male counterparts (t = 5.45; p < 0.001).

## Discussion

This study is to our knowledge the first to evaluate the prevalence of obesity among both men and women in urban and rural Senegal. Moreover, it is also the first study to assess perception of body size in both genders in this country.

In Dakar, the prevalence of general obesity was 9.7%, and that of overweight, 19.2%. These prevalence rates place Dakar among the West African cities that are least affected by problems of excess weight.^31^,^32^ Comparison of our results with those of a study carried out among men and women in Dakar in 2009^11^ suggests that prevalence of general obesity may have increased in the Senegalese capital in five years (17%), but this difference was not statistically significant. However, since 2009, the prevalence of central obesity by WC has increased significantly, by 23% (p < 0.05). In Tessekere, the prevalence of overweight and obesity were 13.3 and 2.8%, respectively. Despite the difficulty of making comparisons with other West African rural areas due to the lack of current data,^10^ these results tend to indicate that Tessekere is also one of the rural areas in the sub-region that is least affected by the obesity epidemic.^33^-^35^

As indicated in the literature on West Africa,^10^ problems of excess weight affect the urban environment more than the rural environment, therefore showing just how environmentally dependent the nutritional transition is in Senegal. In Dakar, the modern lifestyle^36^ is combined with a decrease in physical activity and a higher calorie content diet. In Tessekere, where there is no running water or electricity, a pastoral lifestyle still protects the population from the obesity epidemic, particularly by obliging people to travel long distances daily to feed and water their herds.

However, our results show that such differences between the urban and rural environment may not last, as overweight and obesity rates among women born after the great drought of 1973–1974, hitande bonde [the worst year in Pulaar], are now approaching those of their urban counterparts. The gradual closing of the gap between urban and rural populations is also borne out by results concerning the ideal body size. In the rural environment, the ideal body type for both men and women is in the overweight category, whereas it is in the normal range in Dakar. The social value placed on the overweight body undeniably acts as a factor in the development of excess weight in rural areas.^16^

At the same time, it is important to note the considerable tolerance that both rural and urban Senegalese show toward overweight. At a BMI of 27.5 kg/m², less than 40% of the men in Dakar and Tessekere saw themselves as too fat, compared to 50% of urban women and 30% of rural women. By comparison, in France, for the same BMI, 60% of the men and 85% of women saw themselves as too fat.^29^ Therefore, not only are body weight norms higher in Senegal than in France, but they are also less strict, which can only foster development of the obesity epidemic.^16^ A tightening of these body weight norms is conceivable in the years to come, both pro-actively, through public health messages issued by the Senegalese government, and also through globalisation and the media, which convey beauty standards that emphasise a slimmer body, particularly in the urban environment.^37^,^38^

Our investigation has several limitations. First, the study design was cross-sectional, which does not allow us to explore causation. To overcome this limitation, it would be necessary to conduct a longitudinal study in Dakar in the future. Second, due to insufficient numbers of older adults in the study, we were unable to survey the evolution of body weight after 50 years of age, which should be analysed in the future, given the significant rise in weight-related problems with age, and the aging population on the continent.^39^

## Conclusion

This study shows that the prevalence of obesity is bound to rise quickly among Senegalese women living in a rural environment, partly due to high body weight norms and a large tolerance towards overweight and obesity. To combat problems of obesity in Senegal at present, public health messages should be geared towards the population category most at risk, in other words mature women living in urban areas. However, to limit the scope of the epidemic over the entire country, health centres, which are the only local health structures in rural areas, must begin to raise awareness of the problems that arise with excess body weight.
